# Supplementary material for: Household latrine utilization and associated factors in semi-urban areas of northeastern Ethiopia
Source: PLoS One. 2020 Nov 12;15(11):e0241270. doi: 10.1371/journal.pone.0241270 (PMC7660512; doi:10.1371/journal.pone.0241270)
Supplement: S1 Questionnaire — (DOCX) [file pone.0241270.s002.docx]

## Questionnaire for assessment of Latrine utilization and associated factors

| **Part I (100): - Socio-demographic factors** | | | | | | |
| --- | --- | --- | --- | --- | --- | --- |
| No | Questions | Response options | | | Answer | Skip |
| 101 | Who is the head of the household? | 1. Father 2.Mother 9.other specify------------ | | |  |  |
| 102 | Sex? | 1. Male  2.female | | |  |  |
| 103 | Age? | ----------------year | | |  |  |
| 104 | What is your Religion? | ------------------------ | | |  |  |
| 105 | Marital status? | 1. Single  2. Married  3. Widowed  4. Divorced | | |  |  |
| 106 | What is your Occupation?  (More than one option is possible) | 1. Housewife  2. Daily laborer  3. Government employee  4. Merchant  5. Farmer  9. Other (specify)------------ | | |  |  |
| 107 | What is your education level? | ------------------------ | | |  |  |
| 108 | Total family size of the household? (number) | -------------------------- | | |  |  |
| 109 | Do you have primary or secondary student in your household? | 0. No  1. yes | | |  |  |
| 110 | Do you have under five children in the house? | 0. No  1.Yes | | |  |  |
| **Urban wealth index** | |  | | |  |  |
| 111 | What is the source of your drinking water? (more than one answer is  possible ) | 1.Houseline water  2.Pull and push/sway  common water  3. Bono water  4.Protective pond water  5.un protective pond  water  6.Stream water  7. If others list…….. | | |  |  |
| 113 | Who is the owner of your living house | 1. My own 2. rent house | | |  |  |
| 114 | Does your living house have dividing class? | 0. No  1. Yes | | |  |  |
| 115 | Do you have separated bedroom? | 0. No  1. yes | | |  |  |
| 116 | Do you have separated kitchen? | 0. No  1. yes | | |  |  |
| 117 | From which material your house floor is made? (more than one answer is possible ) | 1. Natural ground  2. Muck/smooth by cows faces  3. Wood  4. Cement  5. If others list …….. | | |  |  |
| 118 | From which material your house roof is made? | 1. Grass/ leaf  2. Corrugated iron | | |  |  |
| 119 | From which material your house wall is made? (more than one answer is possible | 1.Wood but not have mod  2. Wood with mod  3. Wood and cement  4. Blocket  5. If others list…........ | | |  |  |
| 120 | What is your energy source for food cooking? (more than one answer is possible ) | 1. Electric city system  2. Gas /kerosene  3. Wood /leaf  4. Charcoal  5. Animal faeces  6. If other list… | | |  |  |
| 121 | Among the following materials, which one do you own? (more than one answer is possible ) | 1.Radio  2. Television  3. House phone  4. Fridge  5. Chair  6. Table  7. Bed and mattress which made from cotton spring  8. Mobile  9. Cycle  10. Motor cycle  11. Horse’s cart  12. Bajaj/car  13.Bank book  14. If other list …. | | |  |  |
| **Rural wealth index** | | | | |  |  |
| 122 | Among the following materials, which one do you own? (more than one answer is possible ) | | 1.Watch  2. Sofa  3.Chair  4. Table  5.Bed and mattress which made from  6. Cotton spring  7.Horse’s Cart  8. If others specify... | |  |  |
| 123 | Do you have your own farm for the purpose of agriculture/cropping? | | 0. No  1. yes | |  |  |
| 124 | From the following household animal do you have? (more than one answer is possible) | | 1. Ox/ cow  2. Horse/donkey/ mule  3. Goat  4. Sheep  5. Hen  6. Beehive  7. Others................... | |  |  |
| **Part II (200) Environmental factors** | | | | | |  |
| Code | Questions | | | Response options |  | Skip |
| 201 | What kind of latrine facility do you use? ( Observation) | | | 1.Ventilated improved pit latrine (VIP)  2. Pit latrine with slab  3. Pit latrines without a slab or open pit  4. Flush or pour-flush latrine to either: Piped sewer system or Septic tank  5.Open defecation (No facilities bush or field).  9. Other specify--------------- |  |  |
| 202 | Do ≤ 5 children use latrine? | | | 0. No  1. Yes |  |  |
| 203 | What are the reasons for not using the latrine by ≤ 5 children? | | | 1.Floor not safe to stand on  2. Large squat hole  3. Latrine is not clean  4. Unreasonable bad smell  9. Others/specify________ |  |  |
| 204 | Where do you dispose faeces of children who do not start using latrines?(observation) | | | 1. Pit latrine disposal  2. Disposal in the compound  3.Disposal outside the compound  9. Others/specify______ |  |  |
| 205 | What is the condition of latrine? (Observation) | | | 1. Bad latrine: a latrine without superstructure and lack of of privacy during defecation.  2.Fair latrine: latrine having superstructure, without a door (any  Cover) but with a leaking roof and sagging walls.  3. Good latrine: latrine having superstructure with a door (any cover) and possibility of maintaining privacy during defecation. |  |  |
| 206 | Is feces seen around the pit-hole/or on the floor of latrine? ( Observation) | | | 0. No  1.Yes |  |  |
| 207 | Distance of latrine from the house? | | | ----------------meters |  |  |
| 208 | Where is your Latrine location? | | | 1.Inside compound  2.Outside compound  3. No compound  4.Insidehouse  9. Other (specify)………… |  |  |
| 209 | How many years since latrines constructed or service time? | | | ---------year---------month |  |  |
| 210 | Frequency of latrine construction? | | | 1. The 1^st^ latrine  2.The 2^nd^ latrine  3.the 3^rd^ latrine  4.The 4^th^ latrine and above |  |  |
| 211 | Does latrine have Squat hole covered? (observation) | | | 0. No  1. Yes |  |  |
| 212 | Is the slab sealed with mud or cement (observation)? | | | 0. No  1. Yes |  |  |
| 213 | Latrine presence of wall? (Observation). | | | 0. No  1. Yes |  |  |
| 214 | Latrine presence of roof? (Observation). | | | 0. No  1. Yes |  |  |
| 215 | Latrine presence of door? (Observation). | | | 0. No  1. Yes |  |  |
| 216 | Does latrine have superstructure? (Observation). | | | 0. No  1. Yes |  | If No skip to Q218 |
| 217 | Type of material superstructure? (Observation) | | | 1.Wood  2. Wood and mud  3. Grass  4. Cement  5. Plastic  6. Corrugated iron  7. Other specify-------- |  |  |
| 218 | What is the status of latrine? (Observation) | | | 1. No Need Maintenance  2. Need maintenance |  | Q 218 if No need Maintained skip to Q301 |
| 219 | Which parts of the latrine need maintenance? observation (more than one answer is possible) | | | 1.Superstructure  2.floor  3.Roof  4.Wall  5.All  9.Other (specify)----------------- |  |  |
|  | | | | | | |
|  | | | | | | |
| **Part III (300) Behavioral factors** | | | | | |  |
| Code | Questions | | | Response options | Answer | Skip |
| 301 | Cleaning frequency of latrine? | | | 1.weakly  2. Daily  3.When dirt  4.Other (specify)------------------ |  |  |
| 302 | Status of Latrine hygienic conditions? | | | 1. Clean: - no fecal matter in and around the pit latrine, Properly swept.  2. Dirty latrine:-Fecal matter littered in or around the pit latrine not Swept. |  |  |
| 303 | Have you source of information for constructing latrine? | | | 0.No  1.Yes |  | IfQ303 no go to Q305 |
| 304 | What are your reasons to construct latrines? | | | 1.Advice by health extension worker  2. From seeing other  3.Self initiation  4. Impositions from kebele  9.Other (specify)------------- |  |  |
| 305 | Who is responsible for constructing latrines in your household? | | | 1.Men  2.Women  3.Both |  |  |
| 306 | Do you think, In your opinion lack of latrine is considered as cultural taboo? | | | 0.No  1.Yes |  | If No skip to Q 308 |
| 307 | What type of cultural taboo?  (more than one answer is possible) | | | 1.Shame  2.Smell  3.Indignity  4.Flies problem  5. Don’t know  9.Other specify------------------- |  |  |
| 308 | Does latrine have hand washing facility?(Observation) | | | 0.No  1.Yes |  | If No skip to 313 |
| 309 | How close are hand-washing facilities  to the latrine (Observation)? | | | 1.At the entry of the latrine  2. Next to the latrine  3. Inside the house  9.Other specify-------------- |  |  |
| 310 | Is there water in hand washing facility? (Observation) | | | 0. No  1. Yes |  |  |
| 311 | Is there soap near hand washing facility? (Observation) | | | 0. No  1. Yes |  |  |
| 312 | Is there other thing used when you washing your hands? (Observation) | | | 0. No  1. Yes |  |  |
| 313 | What is the effect of open defecation? | | | 1. Causes shame  2. Causes diseases  3. Don’t Know  9. Others (Specify)----------- |  |  |
| 314 | Do you think human feces are a principle source of diarrhoea? | | | 0. No  1. Yes |  |  |

| **Latrine Utilization measurement** | | |  |  |
| --- | --- | --- | --- | --- |
| 001 | Is a household having functional Latrines? (Observation) | 0. No  1. Yes |  |  |
| 002 | Safe disposal of child faeces? (Observation) | 0. No  1. Yes |  |  |
| 003 | No observable faeces in the  Compound? (Observation) | 0. No  1. Yes |  |  |
| 004 | Foot path to the latrine not covered by grass? (Observation) | 0. No  1. Yes |  |  |
| 005 | The latrine is smelly? (Observation) | 0. No  1. Yes |  |  |
| 006 | No spiders weave in squatting hole? (Observation) | 0. No  1. Yes |  |  |
| 007 | Presence of anal cleansing material? (Observation) | 0. No  1. Yes |  |  |
| 008 | Fresh faeces in the squatting hole? (Observation) | 0. No  1. Yes |  |  |
| 009 | The slab is wet? (Observation) | 0. No  1. yes |  |  |
| 010 | utilization of latrine | 0. Not Utilized  1. Utilized |  |  |

**Thank you very much for your participation in this study!**
