# Supplementary material for: Household latrine utilization and associated factors in semi-urban areas of northeastern Ethiopia
Source: PLoS One. 2020 Nov 12;15(11):e0241270. doi: 10.1371/journal.pone.0241270 (PMC7660512; doi:10.1371/journal.pone.0241270)
Supplement: S2 Questionnaire — (DOCX) [file pone.0241270.s003.docx]

ጥያቄዎች በአላንሻ ታዳጊ /የገጠር ከተማ የሽንት ቤት አጠቃቀምና ተዛማጅ ተፅኖዎችን በተመለከተ

| ክፍል አንድ (100): - ማህበራዊና የስነ-ሕዝባዊ ሁኔታዎች | | | | | | | | | | |
| --- | --- | --- | --- | --- | --- | --- | --- | --- | --- | --- |
| ጥያቄ  ቁጥር | | ጥያቄዎች | | | አማረጭ መልሶች | | ምልክት | | የሚዘለል | |
| 101 | | የቤቱ ሃላፊ/አስተዳዳሪ ማን ነዉ? | | | 1.አባት  2. እናት  3.ሌላ ይገለፅ----------------- | |  | |  | |
| 102 | | የቤቱ ባለቤት ፆታ? | | | 1.ወንድ  2.ሴት | |  | |  | |
| 103 | | የቤቱ ባለቤት ዕድሜ ስንት ነዉ ? | | | ----------------በአመት | |  | |  | |
| 104 | | ሀይማኖትዎ ምንድን ነዉ? | | | ----------------------- | |  | |  | |
| 105 | | የቤቱ ባለቤት የጋብቻ ሁኔታ? | | | 1. ያላገባ/ች  2. ያገቡ  3.ባል/ሚስት የሞተችበት/የሞተባት  4. የፈታ/ች | |  | |  | |
| 106 | | የቤቱ ሀላፊ/አስተዳዳሪ ስራ ምንድን ነዉ ?  (ከአንድ በላይ አማራጭ ካለ ይቻላል ) | | | 1. የቤት እመቤት  2. የጉልበት ሰራተኛ  3. የመንግስት ሰራተኛ  4. ነጋዴ  5. ገበሬ  9. ሌላ (ይገለፅ)-------- | |  | |  | |
| 107 | | የቤቱ ሀላፊ/አስተዳዳሪ የትምህርት ደረጃ? | | | -------------------------------- | |  | |  | |
| 108 | | የቤተሰብ አባላት ብዛት? (በቁጥር) | | | --------------------- | |  | |  | |
| 109 | | በቤታችሁ ዉስጥ የመጀመሪያ ደረጃ ወይም ሁለተኛ ደረጃ ተማሪዎች አሉ? | | | 0. የሉም  1. አሉ | |  | |  | |
| 110 | | በቤትዎ ዉስጥ አምስት አመትና በታች ህጻናት አሉ? | | | 0. የሉም  1.አሉ | |  | |  | |
| **የከተማ ሐብት ሁኔታ ጠቋሚ መረጃ** | | | | |  | |  | |  | |
| 111 | | የምትጠቀሙት ዉሃ መገኛው ከየት ነዉ? (ከአንድ በላይ መመለስ ይቻላል) | | | 1.በቤት ዉስጥ ያለ የመስመር ዉሃ  2.በእጅየሚነቀነቅ የጋራ ዉሃ  3.ቦኖዉሃ 4. የተከለለ የጉድጋድ ዉሃ 5.ያልተከለለየጉድጋድ ዉሃ  6. የምንጭ /የወራጅ/ ኩሬ የግድብ ዉሃ  7. ሌላ ካለ ይጥቀሱ ----- - | |  | |  | |
| 112 | | የምትኖሩበት ቤት ባለቤቱ ማን ነዉ? | | | 1. የግልዎ 2. ተከራይተው | |  | |  | |
| 113 | | መኖሪያ ቤታችሁ የተለያየ አገልግሎት እንድሰጥ የተከፋፈለ ነውን? | | | 0. የለም 1. አወ | |  | |  | |
| 114 | | የተለየ የምኝታ ክፍል ለብቻ አለ ወይ? | | | 0. የለም 1. አወ | |  | |  | |
| 115 | | የተለየ የምግብ ማብሰያ /ኩሽና) ክፍል አለ ወይ? | | | 0. የለም 1. አወ | |  | |  | |
| 116 | | የቤቱ ወለል ከምንድን ነዉ የተሰራዉ? (ከአንድ በላይ መመለስ ይቻላል) | | | 1.የተፈጥሮ መሬት /ወለሉ ምንም ነገር የሌለዉ  2. በከብቶች እበት የተሰራ /የተለቀለቀወለል  3. በእንጨት የተሰራ ወለል 4. በሲሚነቶ የተሰራ ወለል  5. ሌላ ካለ ይጥቀሱ….. | |  | |  | |
| 117 | | የቤቱ ጣራ ከምድን ነዉ የተሰራዉ? | | | 1.ከሳር/ከቅጠል2. ከቆርቆሮ | |  | |  | |
| 118 | | የቤቱ ግድግዳ ከምንድን ነዉ የተሰራዉ? (ከአንድ በላይ መመለስ ይቻላል) | | | 1.ከእንጨት ሁኖ ጭቃ የሌለዉ  2. ከእንጨት እና ከጭቃ  3. ከእንጨት እና ከሲሚነቶ  4.ከብሎኬትየተሰራ  5. ሌላ ካለ ይጥቀሱ…… | |  | |  | |
| 119 | | ለምግብ ማብሰያነት የምትጠቀሙበት የሀይል ምንጭ ምንድን ነዉ? (ከአንድ በላይ መመለስ ይቻላል) | | | 1.ኤሌክትሪክ  2.ነጭጋዝ  3.እንጨት/ቅጠል  4.ከሰል  5.ኩበት/በጠጥ  6. ሌላ ካለ ይጥቀሱ…… | |  | |  | |
| 120 | | ክዚህ በታች ከተዘረዘሩት ንብረቶች በቤታችሁ ዉስጥ ያላችሁ የቱ ነዉ? (ከአንድ በላይ መመለስ ይቻላል) | | | 1.ሬደዮ  2.ቴሌቪዥን  3የቤትስልክ  4.ፍሪጅ  5.ወንበር  6.ጠረንጴዛ  7.የጥጥ/የእስቦንጅ/አስፕሪነግ ፍራሽ ያለዉ አልጋ  8. ሞባይል ስልክ  9.ሳይክል  10.ሞተርሳይክል  11.የፈረስጋሪ  12.ባጃጅ/መኪና  13.የባንክቡክ  14. ሌላ ካለ ይጥቀሱ…… | |  | |  | |
| **የገጠር ሐብት ሁኔታ ጠቋሚ መረጃ** | | | | |  | |  | |  | |
| 121 | | ክዚህ በታች ከተዘረዘሩት ንብረቶች በቤታችሁ ዉስጥ ያላችሁ የቱ ነዉ ; (ከአንድ በላይ መመለስ ይቻላል); | | | 1. ሰአት  2. ሶፋ  3. ወንበር  4. ጠረንጴዛ  5.የጥጥ/የእስቦንጅ/አስፕሪነግ ፍራሽ ያለዉ አልጋ  7.የፈረስጋሪ  8. ሌላ ካለይጥቀሱ…… | |  | |  | |
| 122 | | የግላችሁ የሆነ ለምርት/እርሻ የሚሆን መሬት አላችሁ ወይ | | | 0. የለም 1. አወ | |  | |  | |
| 123 | | ከሚከተሉት የቤት እንሰሳት ዉሰጥ የትኛው አላችሁ? (ከአንድ በላይ መመለስ ይቻላል) | | | 1. በሬ፣ላም 2. ፈረስ/አህያ፣በቅሎ 3.ፍየል   4. በግ  5.ዶሮ 6.የንብ ቀፎ  7.ሌላ ካለ ይጥቀሱ…… | |  | |  | |
| **ክፍል ሁለት (200) አካባቢያዊ ምክንያቶች** | | | | | | | | |  | |
| ጥቄ ቁጥር | ጥያቄዎች | | | አማራጭ መልሶች | | | |  | | የሚዘለል |
| 201 | ምን አይነት ሽንት ቤት ነው የምትጠቀሙት? (ተመልከት/ች) | | | 1.ሽታ አልባ/የአየር መዉጫ የተገጠመለት ሽንት ቤት  2. ወለል ያለው ጉድጓድ ሽንት ቤት  3.ወለል የሌለዉ ጉድጓድ ሽንት ቤት  4.ሽንት ቤቱ በዉሃ የሚሰራ እና የቆሻሻ በቱቦ የተገጠመለት/ ሴፕቲክ ታንክ ያለዉ  5. ሽንት ቤት የለም ( ክፍት ቦታ /ሜዳ) ቁጥቋጦ/መስክ ስር  9. ሌላ ይገለፅ--------------- | | | |  | |  |
| 202 | ከ5 ዓመት በታች ያሉ ህፃናት ሽንት ቤት ይጠቀማሉ? | | | 0.አይጠቀሙም  1.አዎ | | | |  | |  |
| 203 | ልጆች ሽን ት ቤት የማይጠቀሙበት ምክንያት ምንድን ነዉ? ( ተመልከት/ች) | | | 1.ወለሉ ለመቆም ስለማይመች  2.ቀዳዳዉ ሰፊ ስለሆነ  3.ንፅህና ስለሌለዉ  4.መጥፎ ሽታ ስላለዉ  9. ሌላ ይገለፅ--------- | | | |  | |  |
| 204 | ሽንት ቤት መጠቀም ያልጀመሩ ልጆችን ሠገራ እንዴት ታስወግዱታላችሁ? | | | 1.ሽንት ቤት ዉስጥ መጣል  2.ግቢ ዉስጥ መጣል  3.ከግቢ ዉጭ መጣል  9. ሌላ ይገለፅ--------- | | | |  | |  |
| 205 | የሽንት ቤቱ ሁኔታ ምን ይመስላል? (ተመልከት/ች) | | | 1.ጥሩ ያልሆነ ሽንት ቤት፡- ከለላ ና ግድግዳ የሌለዉ  2.በቂ/መጠነኛ፡- ከለላያለዉና ምንም አይነት በር የሌለዉ፣ዉሃ የሚያፈስ ቀዳዳ ጣራ ያለዉ  3.ጥሩ፡-ሽንት ቤቱ የቤት ዋና አካል ያለዉ፣በር ያለዉ እና በሚፀዳዱበት ጊዜ ግላዊነት ያለዉ | | | |  | |  |
| 206 | ሠገራ ከጉድጓዱ ቀዳዳ/ ሽንትቤቱ ወለል ላይ አለ ወይ?  (ተመልከት/ች) | | | 0. የለም  1.አዎ | | | |  | |  |
| 207 | ሽንት ቤቱ ከመኖርያ ቤቱ ያለዉ ርቀት ምን ያህል ነዉ | | | ----------------በሜትር | | | |  | |  |
| 208 | ሽንት ቤቱ የሚገኝበት? | | | 1.ከግቢ ዉስጥ  2. ከግቢ ዉጭ  3.ግቢ የለዉም  4. ሌላ ይገለፅ----------- | | | |  | |  |
| 209 | ሽንት ቤቱ ከተሰራ /ካገለገለ ምን ያህል ጊዜ ሆነዉ? | | | -------ዓመት------ወር | | | |  | |  |
| 210 | ሽንት ቤት የሰራችሁ ለስንተኛ ጊዜያችሁ ነዉ? | | | 1. የመጀመሪያ ሽንት ቤት  2.የሁለተኛ ሽንት ቤት  3.የሶስተኛ ሽንት ቤት  ^4. አራተኛ ና በላይ^ ሽንት ቤት | | | |  | |  |
| 211 | የሽንት ቤቱመቀመጫ ቀዳዳ ክዳን አለዉ? ( ተመልከት/ች) | | | 0.የለዉም  1.አዎ | | | |  | |  |
| 212 | የሽንት ቤቱ ወለል ከጭቃ/ ከሲሚንቶ የተለሰነ ነዉ ? ( ተመልከት/ች) | | | 0. የለዉም  1..አዎ | | | |  | |  |
| 213 | ሽንት ቤቱ ግድግዳ አለዉ? ( ተመልከት/ች) | | | 0. የለዉም  1.አዎ | | | |  | |  |
| 214 | ሽንት ቤቱ ጣራ አለዉ? ( ተመልከት/ች). | | | 0. የለዉም  1. አዎ | | | |  | |  |
| 215 | ሽንት ቤቱ በር/መዝጊያ አለዉ? ( ተመልከት/ች) . | | | 0. የለዉም  1. አዎ | | | |  | |  |
| 216 | ሸንት ቤቱ ከለላ አለዉ? ( ተመልከት/ች) | | | 0. የለዉም  1. አዎ | | | |  | | ከሌለዉ ወደ 218 ይዝለሉ |
| 217 | የሽንት ቤቱ ከለላ የተሰራዉ ከምንድን ነዉ? (ተመልከት/ች). | | | 1.ከእንጨት ብቻ  2. ከእንጨትና ጭቃ  3. ከሳር  4. ከሲሚንቶ  5. ከፕላሰቲክ  6. ከቆርቆሮ  9.ሌላ ይገለፅ-------- | | | |  | |  |
| 218 | የመፀዳጃ ቤቱ ሁኔታ ምን ይመስላል? | | | 1.ጥገና አያስፈልገዉም  2.ጥገና ያስፈልገዋል | | | |  | | ካላስፈለገዉ ወደ ቁጥር 301 ይዝለሉ |
| 219 | ጥገና የሚያስፈልገዉ የሽንት ቤት ክፍል የትኛዉ ነዉ? | | | 1.ከለላዉ  2.ወለሉ  3.ጣራዉ  4. ግድግዳዉ  5. ሁሉም  9.ሌላ ይገለፅ---------------- | | | |  | |  |
|  | | | | | | | | | | |
|  | | |  | | |  |  |  |  |  |
|  |  |  |  |  |  |  | | | | |
| **ክፍል ሶስት (300) ከስራ ባህሪ ጋር የተያያዙ ሁኔታዎች** | | | | | | | | |  | |
| ጥያቄ ቁጥር | ጥያቄዎች | | | አማራጭ መልሶች | | | | መልስ | | የሚዘለል |
| 301 | ሽንት ቤቱን በምን ጊዜ ታፀዱታላችሁ? | | | 1.በየሳምንቱ  2.በየቀኑ  3.በቆሸሸ ጊዜ  4.ሌላ ይገለፅ------------------ | | | |  | |  |
| 302 | የሽንት ቤቱ የንፅህና ሁኔታ ምን ይመስላል ? ( ተመልከት/ች) | | | 1.ንፁህ ፡- ጉድጓዱ ያልሞላ ምንም አይነት ሽንትና ሠገራ ወለሉ አካባቢ የሌለዉ፣ በአግባቡ የተፀዳ/የተጠረገ፣ሽታ የሌለዉ  2. ንፁህ ያልሆነ/የቆሸሸ፡- ሠገራና እና ሽንት በአካባዉና በሽንት ቤቱ ወለል ላይ የሚታይና ያልተፀዳ ነዉ | | | |  | |  |
| 303 | ሽንት ቤት ለመስራት የመረጃ ምንጭ አላችሁ? (ተመልከት/ች) | | | 0.የለም  1.አዎ | | | |  | | የለም ካሉ ወደ 305 ይዝለሉ |
| 304 | ሽንት ቤት ለመስራት ያነሳሳችሁ ምክንያት ምንድን ነዉ? | | | 1.በጤና ኤክስቴንሽን በመስማት  2.ሌሎችን በማየት  3.በራስ ተነሳሽነት  4.ከቀበሌ በመገደድ  9.ሌላ ይገለፅ------------ | | | |  | |  |
| 305 | ከቤታችሁ ዉስጥ ሽንት ቤት ለመስራት ሀላፊነት ያለዉ ማን ነዉ? | | | 1.ወንድ  2.ሴት  3.ሁሉም | | | |  | |  |
| 306 | በዕርስዎ አስተያየት ሽንት አለመኖር በባህላችሁ እንደ ነዉር ይቆጠራል ? | | | 0.አደለም  1.አዎ | | | |  | | አይደለም ካለ ወደ308 ይዘለል |
| 307 | ምን አይነት ባህላዊ ነዉር? | | | 1.ያሳፍራል  2.መጥፎ ስም  3.ሽታ  4.የዝንብ ችግር  5. አይታወቅም  9.ሌላ/ ይገለፅ----------------- | | | |  | |  |
| 308 | የሽንት ቤቱ የእጅ መታጠቢያ አለዉ?( ተመልከት/ች) | | | 0.የለም  1. አዎ | | | |  | | የለም ካሉ ወደ 313 ይዘልል |
| 309 | የእጅ መታጠቢያ ዕቃዉ ከሽንት ቤቱ ያለዉ አቀማመጥ ምን ይመስላል? (ተመልከት/ች) | | | 1.ከሽንት ቤቱ መግቢያ  2.ከሽንት ቤቱ ጋር በተያያዘ  3. በቤት ዉስጥ  9.ሌላ ይገለፅፅ---------------- | | | |  | |  |
| 310 | እቃው ውሃ አለው ወይ? (ተመልከት/ች) | | | 0. የለም  1. አለ | | | |  | |  |
| 311 | ከእጅ መታጠቢያ አካባቢ ሣሙና አለ ወይ? (ተመልከት/ች) | | | 0. የለም  1. አለ | | | |  | |  |
| 312 | ከእጅ መታጠቢያ አካባቢ አመድ አለ ወይ? (ተመልከት/ች) | | | 0. የለም  1. አለ | | | |  | |  |
| 313 | ሜዳ ላይ መፀዳዳት ምን አይነት ጉዳት አለዉ? | | | 1. ያሳፍራል  2. በሽታ ያመጣል  3. አይታወቅም  4. ሌላ/ ይገለፅ----------- | | | |  | |  |
| 314 | የሰዉ ሠገራ ዋነኛ የተቅማጥ መነሻ ይሆናል ብለዉ ያስባሉ? | | | 0.የለም  1.አዎ | | | |  | |  |
| **የሽንት ቤት አጠቃቀም መለኪያዎች** | | | | | | | | | | |
| 001 | ሽንት ቤቱ ይሰራል? (ተመልከት/ች) | | | 0. የለም  1. አዎ | | | |  | |  |
| 002 | የህፃናት ሠገራ በአግባቡ ይወገዳል ወይ? (ተመልከት/ች) | | | 0. የለም  1. አዎ | | | |  | |  |
| 003 | በግቢ ዉስጥ ምን አይነት ሠገራ አይታይም ወይ? (ተመልከት/ች) | | | 0. የለም  1. አዎ | | | |  | |  |
| 004 | ወደ የሽንት ቤቱ የሚወስደዉ የእግር መሄጃ መንገድ በሳር ያልተሸፈነ ነዉ? (ተመልከት/ች) | | | 0. የለም  1. አዎ | | | |  | |  |
| 005 | ሽንት ቤቱ ሽታ አለዉ? (ተመልከት/ች) | | | 0. የለም  1. አዎ | | | |  | |  |
| 006 | የሽንት ቤቱ መቀመጫ ቀዳዳ የሸረሪት ድር የሌለዉ መሆኑን ማረጋገጥ? (ተመልከት/ች) | | | 0. የለም  1. አዎ | | | |  | |  |
| 007 | ከተጠቀሙ በኋላ ማፅጃ ነገር አለ ወይ? (ተመልከት/ች) | | | 0. የለም  1. አዎ | | | |  | |  |
| 008 | በሽንት ቤቱ መቀመጫ ቀዳዳ ድር ትኩስ/የእለት ሠገራ አለ ወይ? (ተመልከት/ች) | | | 0. የለም  1. አዎ | | | |  | |  |
| 009 | የሽንት ቤቱ ወለል / መቀመጫዉ አካባቢ እርጥብ ነዉ? (ተመልከት/ች) | | | 0. የለም  1. አዎ | | | |  | |  |
| 010 | የሽንት ቤት አጠቃቀም | | | 0. አይጠቀሙም  1. ይጠቀማሉ | | | |  | |  |

ስለ ተሳትፎዎ በጣም አመሰግናለሁ!!!!
