# Supplementary material for: Household latrine utilization and associated factors in semi-urban areas of northeastern Ethiopia
Source: PLoS One. 2020 Nov 12;15(11):e0241270. doi: 10.1371/journal.pone.0241270 (PMC7660512; doi:10.1371/journal.pone.0241270)
Supplement: Attachment — Submitted filename: PONE-D-20-01859 .pdf [file pone.0241270.s005.pdf]

PLOS ONE

Household Latrine Utilization and Its Association with Household Family Size in Semi-Urban Areas of Alansha, South Wollo, Northeastern, Ethiopia

--Manuscript Draft--

**Manuscript Number:**	PONE-D-20-01859
**Article Type:**	Research Article
**Full Title:**	Household Latrine Utilization and Its Association with Household Family Size in Semi-Urban Areas of Alansha, South Wollo, Northeastern, Ethiopia
**Short Title:**	Household Latrine Utilization
**Corresponding Author:**	Metadel Adane<br>Wollo University<br>Dessie, ETHIOPIA
**Keywords:**	associated factors, cross-sectional study, latrine utilization, semi-urban, Alansha, South Wollo, Ethiopia
**Abstract:**	Background<br>Latrine utilization is the actual behavior that is reflected in a regular practice of using existing latrines for safe excreta disposal. Latrine utilization is a problem in semi-urban areas of developing countries including Ethiopia. Furthermore, the prevalence of latrine utilization among semi-urban residents of Alansha is unknown which needs assessment for the purposes of planning targeted intervention programs. This study is designed to address this gap.<br>Methods<br>A cross-sectional study was conducted from January to March, 2019 among 397 households. Data was collected by trained data collectors via face-to-face interviews and observations using a pretested structured questionnaire. A systematic random sampling method was used for selecting study participant households. The wealth index status of participants was estimated using principal component analysis. A logistic regression model with 95% confidence interval (CI) was employed for data analysis using SPSS version 25.0 software. From the multivariable logistic regression analysis, variables with a significance level at $P < 0.05$ were taken as statistically significant and independently associated with latrine utilization. Model fitness was checked using Hosmer-Lemeshow test and the model is fit at 95.7% (significant at $P = 0.957$).<br>Result<br>A total of 401 households with latrines were assessed for their latrine utilization. The extent of latrine utilization was 285(71.8%). Presence of primary or secondary school student (AOR: 2.33, 95% CI [1.42-3.83]) and household family size of from one to three (AOR: 3.99. 95% CI [1.20-13.24]), number of years since latrine was constructed ≥2 years (AOR: 1.82, 95% CI [1.12-2.95]) and a frequency of daily cleaning of the latrine (AOR: 2.19, 95% CI [1.12-4.28]) were factors associated with latrine utilization.<br>Conclusion<br>Based on the findings, it was concluded that a majority of households utilized the latrine. From this study, a household size from one to three, presence of primary or secondary school student, number of years since construction of latrine of ≥2 years and daily frequency of cleaning of latrines were associated with latrine utilization. It is recommended that promotion of behavioral change toward sustainable and consistent latrine utilization be carried out as an essential step for further improvement of the hygiene and sanitation practices in the community.
**Order of Authors:**	Deres Asnake
	Metadel Adane
**Additional Information:**	
**Question**	**Response**
**Financial Disclosure**	The funders had no role in study design, data collection and analysis, decision to publish, or preparation of the manuscript.

Powered by Editorial Manager® and ProduXion Manager® from Aries Systems Corporation

Enter a financial disclosure statement that describes the sources of funding for the work included in this submission. Review the submission guidelines for detailed requirements. View published research articles from *PLOS ONE* for specific examples.<br><br>This statement is required for submission and **will appear in the published article** if the submission is accepted. Please make sure it is accurate.<br><br>**Unfunded studies**<br>Enter: *The author(s) received no specific funding for this work.*<br><br>**Funded studies**<br>Enter a statement with the following details:<br>• Initials of the authors who received each award<br>• Grant numbers awarded to each author<br>• The full name of each funder<br>• URL of each funder website<br>• Did the sponsors or funders play any role in the study design, data collection and analysis, decision to publish, or preparation of the manuscript?<br>• **NO** - Include this sentence at the end of your statement: *The funders had no role in study design, data collection and analysis, decision to publish, or preparation of the manuscript.*<br>• **YES** - Specify the role(s) played.<br><br>* typeset	
**Competing Interests**<br><br>Use the instructions below to enter a competing interest statement for this submission. On behalf of all authors, disclose any competing interests that could be perceived to bias this work—acknowledging all financial support and any other relevant financial or non-financial competing interests.<br><br>This statement **will appear in the published article** if the submission is accepted. Please make sure it is accurate. View published research articles from *PLOS ONE* for specific examples.	The authors have declared that no competing interests exist

Powered by Editorial Manager® and ProduXion Manager® from Aries Systems Corporation

**NO authors have competing interests**<br><br>Enter: *The authors have declared that no competing interests exist.*<br><br>**Authors with competing interests**<br><br>Enter competing interest details beginning with this statement:<br><br>*I have read the journal's policy and the authors of this manuscript have the following competing interests: [insert competing interests here]*<br><br>* typeset	
**Ethics Statement**<br><br>Enter an ethics statement for this submission. This statement is required if the study involved:<br><br>• Human participants<br>• Human specimens or tissue<br>• Vertebrate animals or cephalopods<br>• Vertebrate embryos or tissues<br>• Field research<br><br>Write "N/A" if the submission does not require an ethics statement.<br><br>General guidance is provided below. Consult the submission guidelines for detailed instructions. **Make sure that all information entered here is included in the Methods section of the manuscript.**	An ethical clearance letter was obtained from the Ethical Review Committee (ERC) of Wollo University College of Medicine and Health Sciences. The respondents were informed about the purpose of the study, and their oral consent was obtained. The respondents' right to refuse or withdraw from participating the interview was fully maintained and the information provided by each respondent was kept strictly confidential.

**Format for specific study types**<br><br>**Human Subject Research (involving human participants and/or tissue)**<br>• Give the name of the institutional review board or ethics committee that approved the study<br>• Include the approval number and/or a statement indicating approval of this research<br>• Indicate the form of consent obtained (written/oral) or the reason that consent was not obtained (e.g. the data were analyzed anonymously)<br><br>**Animal Research (involving vertebrate animals, embryos or tissues)**<br>• Provide the name of the Institutional Animal Care and Use Committee (IACUC) or other relevant ethics board that reviewed the study protocol, and indicate whether they approved this research or granted a formal waiver of ethical approval<br>• Include an approval number if one was obtained<br>• If the study involved *non-human primates*, add *additional details* about animal welfare and steps taken to ameliorate suffering<br>• If anesthesia, euthanasia, or any kind of animal sacrifice is part of the study, include briefly which substances and/or methods were applied<br><br>**Field Research**<br><br>Include the following details if this study involves the collection of plant, animal, or other materials from a natural setting:<br>• Field permit number<br>• Name of the institution or relevant body that granted permission	
**Data Availability**<br><br>Authors are required to make all data underlying the findings described fully available, without restriction, and from the time of publication. PLOS allows rare exceptions to address legal and ethical concerns. See the PLOS Data Policy and FAQ for detailed information.	Yes - all data are fully available without restriction

A Data Availability Statement describing where the data can be found is required at submission. Your answers to this question constitute the Data Availability Statement and **will be published in the article**, if accepted.<br><br>**Important:** Stating 'data available on request from the author' is not sufficient. If your data are only available upon request, select 'No' for the first question and explain your exceptional situation in the text box.<br><br>Do the authors confirm that all data underlying the findings described in their manuscript are fully available without restriction?	
**Describe where the data may be found in full sentences. If you are copying our sample text, replace any instances of XXX with the appropriate details.**<br><br>• If the data are **held or will be held in a public repository**, include URLs, accession numbers or DOIs. If this information will only be available after acceptance, indicate this by ticking the box below. For example: *All XXX files are available from the XXX database (accession number(s) XXX, XXX.).*<br>• If the data are all contained **within the manuscript and/or Supporting Information files**, enter the following: *All relevant data are within the manuscript and its Supporting Information files.*<br>• If neither of these applies but you are able to provide **details of access elsewhere**, with or without limitations, please do so. For example:<br><br>*Data cannot be shared publicly because of [XXX]. Data are available from the XXX Institutional Data Access / Ethics Committee (contact via XXX) for researchers who meet the criteria for access to confidential data.*<br><br>*The data underlying the results presented in the study are available from (include the name of the third party*	All relevant data are within the manuscript and its Supporting Information files

and contact information or URL).<br>• This text is appropriate if the data are owned by a third party and authors do not have permission to share the data.<br><br>* typeset	
Additional data availability information:	

Manuscript

Click here to access/download;Manuscript;Final version-Latrine Utilization Manuscript-PONE-D-.doc

Household Latrine Utilization and Its Association with Household Family Size in Semi-Urban Areas of Alansha South Wollo, Northeastern, Ethiopia

Deres Asnake[1], Metadel Adane[2*]

[1]Quality Improvement Unit, Woldia General Hospital, Woldia, Ethiopia.

[2]Department of Environmental Health, College of Medicine and Health Sciences, Wollo University, Dessie, Ethiopia.

***Corrsponding author: metadel.adane2@gmail.com.**

Abstract

Background

Latrine utilization is the actual behavior that is reflected in a regular practice of using existing latrines for safe excreta disposal. Latrine utilization is a problem in semi-urban areas of developing countries including Ethiopia. Furthermore, the prevalence of latrine utilization among semi-urban residents of Alansha is unknown which needs assessment for the purposes of planning targeted intervention programs. This study is designed to address this gap.

Methods

A cross-sectional study was conducted from January to March, 2019 among 397 households. Data was collected by trained data collectors via face-to-face interviews and observations using a pretested structured questionnaire. A systematic random sampling method was used for selecting study participant households. The wealth index status of participants was estimated using principal component analysis. A logistic regression model with 95% confidence interval (CI) was employed for data analysis using SPSS version 25.0 software. From the multivariable logistic regression analysis, variables with a significance level at $P < 0.05$ were taken as statistically significant and independently associated with latrine utilization. Model fitness was checked using Hosmer-Lemeshow test and the model is fit at 95.7% (~~significant at~~ $P = 0.957$).

Result

A total of 401 households with latrines were assessed for their latrine utilization. The extent of latrine utilization was 285(71.8%). Presence of primary or secondary school student (AOR: 2.33, 95% CI [1.42-3.83]) and household family size of from one to three (AOR: 3.99. 95% CI [1.20-

13.24]), number of years since latrine was constructed ≥2 years (AOR: 1.82, 95% CI [1.12-2.95]) and a frequency of daily cleaning of the latrine (AOR: 2.19, 95% CI [1.12-4.28]) were factors associated with latrine utilization.

Conclusion

Based on the findings, it was concluded that a majority of households utilized the latrine. From this study, a household size from one to three, presence of primary or secondary school student, number of years since construction of latrine of ≥2 years and daily frequency of cleaning of latrine were associated with latrine utilization. It is recommended that promotion of behavioral change toward sustainable and consistent latrine utilization be carried out as an essential step for further improvement of the hygiene and sanitation practices in the community.

Key words: associated factors, cross-sectional study, latrine utilization, semi-urban, Alansha, South Wollo, Ethiopia

Background

It is estimated that one billion people worldwide do not have access to any facilities at all for excreta disposal, and instead practice open defecation (O[illegible]Lack of sanitation facilities compels people to practice open defecation, which increases the risk of transmission of diseases. In 201[illegible] 15% of the population still practiced open defecation (UNICEF and WHO, 2014[illegible]he highest proportions of populations practicing open defecation were found in South Asia and Sub Saharan Africa (UNICEF and WHO, 2015[illegible]n Ethiopia up to 60% of the current disease burden is attributable to poor sanitation. Latrine facility coverage has been increasing since the Health Extension Program started, but there has been less attention to quality and utilization of latrine facilities in rural areas compared to urban areas of Ethiopia (Yimam et al., 2014)[illegible]

Lack of latrine utilization has been due mainly to community attitude, a lack of understanding of the benefits of latrine use and the nature of the work the community engaged i[illegible]A study in Southern Ethiopia showed latrine status and utilization in the rural community of Chencha district was very low and that attention was needed to promote hygiene and sanitation behavior in the community. Problems related to latrine utilization included lack of a functional latrine, latrines located a long distance away from areas where farmers work, and latrines lacking a superstructure (Koyra et al., 2017). Continuing needs include provision of sanitation facilities in all parts of Ethiopia with interventions of the Health Extension Program and continued investments to increase access to safe water and improved sanitation (MOH, 2010)[illegible]

A study conducted in Amhara, Ethiopia, found that latrine utilization was associated with education, relative wealth, urban residence and history of travel (Sinha, 2017). In Denbia District

a study found that integration of strategies to promote hygiene behavioral change with construction of sanitation facilities is crucial; and that a long-established habit and comfort with open defecation was the main reason ~~of~~ 60.4% of adults ~~for not using~~ a latrine (Yimam et al., 2014). Ethiopia has made a great effort to increase the sanitation coverage of the country and the reduction rate of open defecation has been remarkable; a 25% reduction per decade for the years 1990-2015 (from 92% in 1990 to 29% in 2015) (UNICEF and WHO, 2015).

In spite of this progress, Ethiopia is still among the top ten open-defecating countries of the world, a group that includes five in Africa (Nigeria, Ethiopia, Sudan, Niger and Mozambique), and five in Asia. The ~~recent data from the~~ Mini Ethiopia Demographic and Health Survey (EDHS) indicate that more than half (55%) of households (56.7% in rural and 4.4% in urban areas) of Ethiopia have access only to a non-improved sanitation facility (pit latrine without slab or pit latrine) (CSA and ICF-International, 2014).

Overall, 6% of Ethiopian households use improved toilet facilities (16% in urban areas and 4% in rural areas). More than half (56%) of rural households use unimproved toilet facilities. In urban households, more than one-third (35%) of toilet facilities are shared with other households, whereas only 2% of rural households share their toilet facilities with others. One in three households in Ethiopia have no toilet facility (39% in rural areas and 7% in urban areas) (CSA and ICF-International, 2016).

These statistics point up the need for this study in order to establish baseline information on latrine utilization and to identify factors affecting the utilization of latrine in semi-urban areas such as Alansha. The World Health Organization's Sustainable Development Goal (SDG) 6 has

a target of achieving access to basic sanitation for all. An understanding of the prevalence of latrine utilization in previously-unstudied areas such as Alansha is needed for the purposes of planning targeted intervention programs to improve sanitation toward this SDG goal. This study was designed to address this need.

Materials and Methods

Study setting

This study was conducted in semi-urban area of Alansha is located in Kutaber Woreda of South Wollo Zone, 12 km from Dessie City. Agriculture is the area's main economic activity. It has a total population of 8,907, including 4426 (49.7%) men and 4481(50.3%) women, and a total of 1,845 households. Two elementary schools and one health post are found in the area. Alansha is situated on a plateau and some sloping areas in a mountainous region that runs north to south. Latrine coverage in the areas is 99% (Semi-urban Alansha *kebele* Administration, unpublished written document data, 2018).

Study design and outcome variable

A cross-sectional study was conducted in the semi-urban of Alansha from January to March, 2019. The outcome variable of this study was latrine utilization, denoted as yes (1) for utilized or no (0) for not utilized. Using the outcome variable of utilized latrine, we estimated the prevalence of latrine utilization among the participating respondents. Latrine utilization was identified using World Health Organization (WHO) and Joint Monitoring Program (JMP) "signs of use" by questioning the respondents about signs of latrine utilization such as a household having a functional latrine, children's feces being safely disposed of, no observable feces in the

compound and at least one observable sign of use (e.g., foot path to the latrine not covered by grass, latrine odor, lack of spiderweb in squatting hole, presence of anal cleansing material, fresh feces in the squatting hole, or a wet slab).

Sample size determination and sampling procedures

Sample size was determined by using a formula for single population proportion by applying the following assumptions. Assumptions were - 5% marginal error, 95% confidence level with the proportion (P= 57.3%) of latrine utilization taken from a study conducted in Tigray Gulomekada District (Debesay *et al*., 2010).

$$n = \frac{\left(\frac{Z\alpha}{2}\right)2 * p(1-p)}{W2}$$

p = Proportion of latrine utilization (57.3%)

w = Marginal error, Zα/2=1.96

$n = (1.96)^2\ 0.573(1-0.573)/0.05^2$

n = (3.8416) 0.573(0.427)/0.0025

ni (initial sample size) = 0.9399/0.0025=375.9 =376

Since the sample size was less than 10,000 at 8,907, a correction formula was used.

As a result, using correction formula $nc = \frac{ni}{1+(ni-1/N)} = 361$

Where:

nc = sample size correction, *ni* = initial sample size, *N* = total population

Adding 10% non-respondent rate, the final sample size became 401.

The semi-urban area of Alansha had one *kebel*e with 1,845 total households, including a total population 8,907 from which a final sample size of 401 was included. The source populations of this study were all people in the semi-urban area of Alansha and the study population was those households selected from residents of semi-urban Alansha. Systematic random sampling was used to find households; total households divided by final sample size gave a sampling interval of 5. Then, data were collected in every fifth interval number of houses.

Inclusion and exclusion criteria

All households with a functional latrine during the study period were included. People who were less than 18 years old during the data collection period were excluded as study participants.

Operational definitions

Semi-urban: A partially urban, partially rural area that was not within a municipality; where the population of city residents was 2000 or more; where it was estimated that there was potential to earn income, on average, other than land lease sale, of 25,000 Birr or mo[illegible]where 20% of city residents engaged in an occupation other than agriculture; that may serve as a development center; that had good transportation (FDRE, 2009).

Functional latrine: Latrine that provided services at the time of data collection even if the latrine required maintenance.

Safe disposal of child feces: Children's feces disposed of in a toilet, not in the open.

Clean latrine: Pit was not full, had no fecal matter around the pit was properly swept and did not have bad smell at time of data collection.

Open defecation: **S**elf-reported behavior, including defecating in fields, bushes, forests, open bodies of water, or other open spaces.

Data collection and data quality assurance

A structured questionnaire was developed for data collection. Data were collected by face-to-face interviews and observation using structured and pretested questionnaires. The questionnaires were first prepared in English and translated to Amharic, and then re-translated back to English to ensure consistency. Four data collectors who are Environmental Healt[illegible] professionals collected the data via interviews with heads of households and observation of the latrine.

A pretest of the questionnaire was conducted with 5% of sample size~~;~~ (20 of 401 questionnaires) to check the consistency and clarity of the questions. It was undertaken on 20 households that were not included in the study before the actual data collection period. The aim was to determine if there were any difficulties filling the questionnaire, challenges in interviewing, or misunderstanding of the questions by enumerators. During data collection, one supervisor collected the questionnaires from each enumerator on a daily basis, checking the consistency and the completeness of the completed questionnaire on the spot.

The principal investigator provided ~~one day's~~ trainin[illegible]r data collectors and supervisors before actual data collection took place. The training was focused on how to fill the questionnaire and how to approach the respondents. Field supervision and daily meetings solved any problems that came up during data collection to ensure the quality of data collection. To check the quality of the data, 10% of entered data (40 questionnaires) was re-entered to check the consistency of originally entered data.

Independent variables

Eight socio-demographic variables were considered in this analysis as potential confounders: marital status, educational status, presence of primary or secondary school student in the household, occupation, household income, presence of ≤5 children and family size. Twelve environmental variables were included in this study such as distance to latrine, year of latrine service construction, presence of squatting hole cover, presence of a door, frequency with which latrine has been constructed, maintenance (latrine needing maintenance or not needing maintenance during data collection), presence of roof, wall and superstructure, latrine with slab or without slab. Types of latrine were measured by data enumerators using direct observation (VIP latrine, pit latrine with slab, and pit latrine without slab) and presence of feces around pit hole/floor of latrine.

Fourteen behavioral variables were included, such as frequency of cleaning and hygienic condition of latrine, socio-cultural taboos, source of information on how to construct a latrine (health extension worker, observation of others), self-initiated construction or imposed by *kebele*, presence of handwashing facility (with water, soap, and/or other [ash[illegible]

Data management and analysis

All field questionnaires were first checked, coded and entered in to EpiData version 3.1 and then exported into SPSS version 2[illegible] for data cleaning and analysis. The economic status of participants was calculated by using a wealth index score. The score was derived using principal component analysis (PCA). Wealth index was calculated by national wealth quintiles compiled by assigning the household score to each usual (de jure) household member, ranking each person

in the household population by her or his score, and then dividing the distribution into five equal categories that are poorest, second, middle, fourth and highest. ~~Multicollinearity was checked using standard error with cut point 2 and the model fitness using Hosmer-Lemeshow test was significant at *P* = 0.957.~~

Binary logistic regression model was fitted to assess factors associated with latrine utilization. Bivariate analysis (crude odds ratio [COR] with 95% CI) was used to assess the crude association and to select important variables to be included in the final model. From the bivariate analysis, $p<0.25$ was selected in multivariable logistic regression model analysis. Finally, multivariable-logistic regressions (adjusted odds ratio [AOR]) with 95% CI was used to control potential confounders and to identify independent predictors of latrine utilization. From the adjusted analysis, a significance level of $P < 0.05$ was used to decide factors significantly associated with latrine utilization. Finally, the result was presented in text, tables and graphs.

Ethical [illegible]nsideration

An ethical clearance letter was obtained from the Ethical Review Committee (ERC) of Wollo University College of Medicine and Health Science[illegible]he respondents were informed about the purpose of the study, and their oral consent was obtained. The respondents' right to refuse or withdraw from participating the interview was fully maintained and the information provided by each respondent was kept strictly confidential.

Results

Socio-demographic characteristics

~~In this study of a total of 401 participants,~~ 397 were involved with a response rate of 99% respondents. ~~Out of those respondents, 285~~(71.8%) utilized latrines~~,~~ whereas 112(28.2%) did not utilize latrines. ~~Of the 397 total respondents,~~ 275(69.3%) were female and the rest were male 122(30.7%). A large proportion of respondents 163 (41.1%) were aged 41-60 years. The number of respondents ~~of Muslim religion was~~ 379 (95.5%), and ~~of~~ other religions were 18 (4.5%). The bivariate analysis of other socio-demographic factors with latrine utilization are summarized in Table 1.

Environmental Characteristics with Latrine Utilization

Among households included in the study were found 86 (21.7%) pit latrines with slab and 311 (78.5%) pit latrines without slab. In 43.8% of households it had been ≥2 years since the latrine was constructed. Households with a latrine slab sealed with mud/cement was (21.7%). Bivariate analyses are summarized in Table 2.

Behavioral Characteristics with Latrine Utilization

Cleaning frequency of the latrine among 30.2% of households was rarely, and 24.7% daily. The hygienic condition of the latrine was clean in 17.15% of households and dirty in 82.9%. Bivariate analysis summarized in Table 3.

Factors associated in multivariable analysis

Of socio-demographic factors, the presence of primary or secondary school student and household size were significantly associated with latrine utilization. Households with presence of a primary or secondary school student were 2.33 times more likely to ~~have utilized~~ a latrine than those without a student (AOR: 2.33, 95% CI [1.42-3.3.83]). Households of a size from one to three family members were 3.99 times more likely to have utilized their latrine compared with households of greater than six members (AOR: 3.99, 95% CI [1.20-13.24]) (Table 4).

Among environmental factors, number of years since the latrine was constructed was a significant factor for latrine utilization. Households where it had been ≥ 2 years since the latrine was constructed were 1.82 times more likely to have utilized the latrines than households with latrines constructed less than two (<2) years (AOR: 1.82, 95% CI [1.12-2.95]) ~~previously~~ (Table 4).

Among behavioral factors, frequency of latrine cleaning was a significant variable~~,~~ after adjusting for other confounder variables in the multivariable logistic regression analysis. Households that cleaned the latrine daily were 2.17 times more likely to utilize their latrine ~~as~~ compared ~~with households~~ who cleaned their latrine rarely (AOR: 2.19, 95% CI [1.12-4.28]) (Table 4).

Discussion

This study used a cross-sectional design to assess latrine utilization status and associated factors in the semi-urban area of Alansha, south Wollo zone, Northeastern Ethiopia. The study found that prevalence of latrine utilization was 71.8% (95% CI 67.5-71.6). In this study, household size of one to three peoples, presence of primary or secondary school student, more than two years since latrine had been constructed and daily cleaning of latrine were significantly associated with latrine utilization.

The finding of latrine utilization in this study was lower than in a study done in Wondo Gene[illegible] (Ashenafi et al., 2018) and Hotesa Arisi zone (Damtew, 2017), but higher than studies done in various other areas of Ethiopia such as Anded (Chanie et al., 2016), Denbia (Yimam et al., 2014), Chencha (Koyra et al., 2017) and Enderta (Belachew et al., 2018). It might be that since Alansha is a semi-urban area, the more urban communities may have had a better awareness of sanitation and hygiene practices compared with more rural areas; communities in semi-urban areas are more or less literate, a lot of government employee live there, and their urban lifestyle is a little different from that in rural areas, all of which may positively influence latrine utilization.

Among socio-demographic factors, based on the result of multivariable analysis, the finding of family size from one to three significantly associated with latrine utilization is similar to a study in Southeast zone of Tigray (Gebremedhin et al., 2018) and in Hawassa (Tulu et al., 2017), which found a family of small size utilized the latrine more than a family of larger size. This finding might be due to the lower number of people in contact with the latrine, making the latrine

more likely to be clea[illegible]o utilization may increase, while a large family size creating many contacts with latrine might make the latrine more likely to be dirty and therefore decrease utilization of latrin[illegible]

Presence of primary or secondary school student finding is similar to the study in Hullet Eju Enessie (64.7%) **(**Anteneh and Kumie, 2010) and Denbia in Gonder (39.6%) (Yimam et al., 2014). The presence of a primary or secondary school student positively favored an increase in of latrine utilization in the home environment (Robinah et al., 2008). This might be due to the fact that secondary school students were more exposed to hygiene information in the school environment and therefore their presence positively favored latrine utilization in the home environment. The district reported (personal communication) that the health extension program was closely linked to the promotion of school health, which was an additional opportunity for students to learn healthy lifestyles.

Of environmental factors, the finding that households in which it had been two years or more since the latrine was constructed were 1.82 times more likely to have utilized their latrine compared with those in which it had been less than two years since the latrine was constructed which is consist with a study in Awabale Gojjam Molla (Chanie et al., 2016), in Chencha district and in Dilla town (Gezu et al., 2015). A study done in Chencha (Koyra et al., 2017) and in Wondo Genet (Ashenafi et al., 2018) in Ethiopia showed that households with more recently (<2 years) constructed latrines were 78% less likely to use the latrine than households with latrines constructed >2 years ago.

The study conducted in India supports this result that more years since a latrine had been constructed increased the utilization of the latrine. This may be due to the fact that attainment of

behavioral change in a community may take some period of time. Hence, education, support and monitoring of sanitary activity all need to be maintained until community behavioral change is observed rather assuming such change will occur as a result of information provided only in short intermittent campaigns.

From the behavioral factors, the finding that daily cleaning of the latrine was significantly associated with latrine utilization is similar to a study in Wondo Genet (Ashenafi et al., 2018), Denbia, Hetosa Woreda in Arsi zone (Damtew, 2017), Michew in (Gebremedhin et al., 2018) and Anded (Chanie et al., 2016). Our finding that daily latrine cleaning helped latrine utilization was consist with a study in a study in Kenya (Waithaka and Racheal, 2015). This might be due to frequent cleaning preventing users coming in contact with faeces and flies and, perhaps more crucially, unsanitary conditions and odour, all of which may deter people from using them. The limitation of this study was the cross-sectional designs which measured the exposure and outcome at the same time.

Conclusion

~~Based on the findings, it was concluded that the majority of households utilized their latrines, and that household size of one to three, presence of primary or secondary school student, two years or more since the latrine was constructed and a daily frequency cleaning of the latrine were significantly associated with latrine utilization~~. Further observational research triangulated with qualitative study should be conducted to provide more strong evidence of factors associated with latrine utilization.

List of Abbreviations

AOR, adjusted odds ratio; CI, confidence interval; COR, crude odds ratio; EDHS, Ethiopia Demographic and Health Survey; MDGs, Millennium Development Goals; MOH, Ministry of Health; NGOs, non-governmental organizations; NODF, none open defecation free; ODF, open defecation free; UN, United Nations; UNICEF, United Nations Children's Fund; VIP, ventilated improved pit latrine.

Acknowledgments

We acknowledge Kutaber *Woreda*, Alansha *kebele* and South Wollo Zone municipality administrators for their kind assistance in providing necessary information and support during data collection. ~~We appreciate the support of Amhara Regional Government Health Bureau by providing for funds for this stud~~ We also thank the respondents, data collectors, and supervisors for their dedication and commitment during the study. We also highly acknowledge Lisa Penttila for the language editing of this manuscript.

References

ANTENEH, A. & KUMIE, A. 2010. Assessment of the impact of latrine utilization on diarrhoeal diseases in the rural community of Hulet Ejju Enessie Woreda, East Gojjam Zone, Amhara Region. *Ethiopian Journal of Health Development,* 24.

ASHENAFI, T., DADI, A. F. & GIZAW, Z. 2018. Latrine utilization and associated factors among Kebeles declared open defecation free in Wondo Genet district, South Ethiopia, 2015.

BELACHEW, A. B., ABRHA, M. B., EBREZGI, Z. A. G. & TEKLE, D. Y. 2018. Availability and utilization of sanitation facilities in Enderta district, Tigray, Ethiopia. *J Prev Med Hyg,* 59**,** DOI: 10.15167/2421-4248/jpmh2018.59.3.826 PMCID: PMC6196372.

CHANIE, T., GEDEFAW, M. & KETEMA, K. 2016. Latrine utilization and associated factors in rural community of Aneded district, North West Ethiopia, 2014. *J Community Med Health Educ,* 6**,** 1-12.

CSA & ICF-INTERNATIONAL 2014. Central Statistical Agency, ICF-International. Mini Ethiopia Demographic and Health Survey 2014. Addis Ababa, Ethiopia, and Calverton, Maryland, USA: Central Statistical Agency [Ethiopia] and ORC Macro.

CSA & ICF-INTERNATIONAL 2016. Central Statistical Agency, ICF-International. Ethiopia Demographic and Health Survey 2011. Addis Ababa, Ethiopia, and Calverton, Maryland, USA: Central Statistical Agency [Ethiopia] and ORC Macro.

DEBESAY, N., INGALE, L., GEBRESILASSIE, A., ASSEFA, H. & YEMANE, D. 2010. Latrine Utilization and Associated Factors in the Rural Communities of Gulomekada District, Tigray Region, North Ethiopia, 2013: A Community Based Cross-Sectional Study. J Community Med Health Educ 5: 338. *Ethiopia, still the national open defecation rate in*.

GEBREMEDHIN, G., TETEMKE, D., GEBREMEDHIN, M., KAHSAY, G., ZELALEM, H., SYUM, H. & GERENSEA, H. 2018. Factors associated with latrine utilization among model and non-model families in Laelai Maichew Woreda, Aksum, Tigray, Ethiopia: comparative community based study. *BMC research notes,* 11**,** 586.

GEZU, A., ESHETU, M. & V., P. K. 2015. Assessing Privy (Latrine's) Utilization and Associated Factors among Households in Dilla Town, Ethiopia, (July). *International Journal of Health Sciences and Research*

KOYRA, H. C., SORATO, M. M., UNASHO, Y. S. & KANCHE, Z. Z. 2017. Latrine utilization and associated factors in rural community of Chencha District, Southern Ethiopia: a community based cross sectional study. *American J Pub Health Res,* 5**,** 98-104.

MOH 2005. Federal Democratic Republic of Ethiopia Ministry of Health national hygiene and sanitation strategy for to enable 100% adoption of improved hygiene and sanitation.

MOH 2010. Health Sector Development Program: 2010/11 -2014/15. Federal Democratic Republic of Ethiopia Ministry of Health (Vol. IV).

NEGUSE, D., LALIT, I., AZEB, G., HURUY, A. & DEJEN, Y. 2013. Latrine Utilization and Associated Factors in the Rural Communities of Gulomekada District, Tigray Region, North Ethiopia, 2013: A Community Based Cross-Sectional Study. *Journal of Community Medicine & Health Education* 5**,** DOI: 10.4172/2161-0711.1000338.

ROBINAH, D., KADDU, J. & MANGEN, W. 2008. Intestinal helminths in Luweero district, Uganda. *Afr Health Sci,* 8**,** 90-6.

SINHA, A. 2017. *Assessing latrine use in low-income countries: a field study in rural India.* London School of Hygiene & Tropical Medicine.

TULU, L., KUMIE, A., HAWAS, S. B., DEMISSIE, H. F. & SEGNI, M. T. 2017. latrine utilization and associated factors among kebeles implementing and non implementing Urban Community led Total sanitation and hygiene in Hawassa town, Ethiopia. *African Journal of Environmental Science and Technology,* 11**,** 151-162.

UNICEF 2012. *The State of the World's Children 2012. Children's in an Urban World,* United Nations Children's Fund. United Nations, New York.

UNICEF & WHO 2014. Joint Monitoring Programme for Water Supply and Sanitation : Jenave: WHO and UNICEF, 2014. Jenave.

UNICEF & WHO 2015. Joint Monitoring Programme for Water Supply and Sanitation: Jenave: WHO and UNICEF, 2014. Jenave.

WAITHAKA & RACHEAL, W. 2015. *Latrine use and associated factors among rural community members in Samburu east sub-county, Samburu county, Kenya.* Kenyatta University.

YIMAM, Y. T., GELAYE, K. A. & CHERCOS, D. H. 2014. Latrine utilization and associated factors among people living in rural areas of Denbia district, Northwest Ethiopia, 2013, a cross-sectional study. *The Pan African medical journal,* 18.

Table 1: Socio-demographic characteristics and bivariate analysis with latrine utilization in semi-urban area of Alansha, South Wollo, January to March, 2019

Variables		Latrine utilization (N=397)		COR (95%CI)	*p*-value
	Frequency (%)	Utilized (n=285)	Not utilized (n=112)		
**Head of household**					
Mother	311 (78.3)	62	24	1.02(0.59-1.73)	0.943
Father	86 (21.7)	223	88	1	
**Sex**					
Male	122(30.7)	91	31	1.23(0.76-1.99)	0.409
Female	275(69.3)	194	81	1	
**Age (years)**					
18-40	100 (25.2	67	33	1	
41-60	163(41.1)	123	40	1.51(0.87-2.62)	0.138*
61-80	76(19.1)	52	24	1.06(0.56-2.02)	0.842
>81	58(14.6)	43	15	1.4(0.68-2.9)	0.348
**Occupation**					
Housewife	166(41.8)	126	40	1.32(0.80-2.20)	0.270
Day laborer	32(8.1)	18	14	0.54(0.25-1.19)	0.126*
Government employee	25(6.3)	16	9	0.75(0.31-1.83)	0.526
Merchant	29(7.3)	23	6	1.62(0.62-4.25)	0.330
Farmer	145(36.5)	102	43	1	1
**Educational status**					
Unable to read and write	205(51.6)	149	56	1	
Able to read and write	70 (17.6)	49	21	0.89(0.48-1.59)	0.666
Primary	59 (14.9)	41	18	0.86(0.45-1.630	0.631
Secondary	39 (9.8)	27	12	0.85(0.40-1.78)	0.660
Post-secondary	24 (6.0)	19	5	1.42(0.51-4.01)	0.498
**Household size (persons)**					
1-3	365(91.9)	267	98	3.81(1.18-12.30)	0.025*
4-6	20(5.0)	13	7	2.60(0.60-11.31)	0.203*
>6	12 (3.0)	5	7	1	1
**Presence of primary or secondary school**					

**student in household**					
No	99(24.9)	57	42	1	
Yes	298(75.1)	228	70	2.40(1.49-3.88)	0.000*
**Presence of under-five children in household**					
No	202(50.9)	177	74	1.05(0.68-1.63)	0.826
Yes	195(49.1)	108	38	1	1
**Wealth index**					
Lowest	104(26.2)	69	35	1	
Second	115(29.0)	85	30	1.44(0.80-2.57)	0.222*
Middle	80(20.2)	59	21	1.43(0.74-2.71)	0.280
Fourth	55(13.9)	41	14	1.49(0.71-3.08)	0.288
Highest	43(10.8)	31	12	1.31(0.60-2.86)	0.497

1 Reference category; * variables from bivariate analysis of $p < 0.25$

Table 2: Environmental characteristics and bivariate analysis with latrine utilization in semi-urban areas of Alansha, South Wollo, January to March, 2019

**Variables**	**Latrine utilization (N=397)**			**COR (95% CI)**	***p*-value**
	**n (%)**	**Utilized (n = 285)**	**Not utilized (n = 112)**		
**Type of latrine**					
Pit latrine with slab	86(21.7)	66	20	1.39(0.80-2.42)	0.250*
Pit latrine without slab	311(78.3)	219	92	1	1
**Reasons for children under five years not using latrine**					
Floor not safe to stand on	200(50.4)	137	63	1	
Large squat hole	144(36.2)	108	36	1.38(0.85-2.23)	0.190*
Latrine not clean	53(13.4)	40	13	1.42(0.71-2.83)	0.326
**Distance of latrine from the house in meters**					
<6	14(3.5)	9	5	0.57(0.17-1.98)	0.380
6-50	321(80.9)	229	92	0.79(0.42-1.49)	0.474
>50	62(15.6)	47	15	1	1
**Latrine location**					
Inside compound	124(31.2)	84	40	0.96(0.52-1.75)	0.880
Outside compound	193(48.6)	146	47	1.41(0.79-2.51)	0.240*
No compound	80(20.2)	55	25	1	1
**Years since latrine constructed**					
<2	223(56.2)	149	74	1	
≥2	174(43.8)	136	38	1.78(1.23-2.80)	0.013*
**Frequency of latrine construction**					
First time	93(23.4)	64	29	1	
Second time	187(47.1)	133	54	1.12(0.65-1.92)	0.691
Third time	67(16.9)	53	14	1.72(0.82-3.56)	0.150*
Fourth or above times	50(12.6)	35	15	1.06(0.50-2.23)	0.884
**Presence of wall**					

No	99(24.9)	57	42	1	
Yes	298(75.1)	228	70	2.4(1.49-3.88)	0.001*
**Presence of roof**					
No	240(60.5)	170	70	1	1
Yes	157(39.5)	115	42	1.12 (0.72-1.77)	0.601
**Presence of door**					
No	103(25.9)	62	41	1	1
Yes	294(74.1)	223	71	2.08(1.29-3.35)	0.003*

1, Reference category; * Variables from bivariate analysis of $p<0.25$

Table 3: Behavioral characteristics and bivariate analysis with latrine utilization in semi-urban of Alansha, South Wollo, January to March, 2019

Variables	Latrine utilization (N=397)			COR (95% CI)	*p*-value
	n (%)	Utilized (n=285) n (%)	Not Utilized (n=112) n (%)		
**Frequency of latrine cleaning**					
Weekly	98(24.7)	64	34	0.83(0.47-1.48)	0.545
Daily	96(24.2)	78	18	1.93(1.01-3.67)	0.045*
When dirty	83(20.9)	60	23	1.16(0.62-2.15)	0.632
Other (rarely)	120(30.2)	83	37	1	1
Information received about constructing latrine					
No	102(25.7)	62	40	1	1
Yes	295(74.3)	223	72	2.0(1.24-3.22)	0.005*
**Perceived reasons to construct latrine**					
Advice by health extension worker	248(62.5)	177	71	1.13(0.38-3.39)	0.823
From seeing others	40(10.1)	26	14	0.84(0.24-2.92)	0.789
Self-initiated	93(23.4)	71	22	1.47(0.46-4.68)	0.517
Imposition from *kebele*	16(4.0)	11	5	1	1
**Presence of handwashing facility**					
No	154(38.8)	111	43	1.02(0.65-1.60)	0.919
Yes	243(61.2)	174	69	1	1
**Presence water in handwashing facility**					
No	265(66.8)	184	81	1	
Yes	132(33.2)	101	31	1.43(0.89-2.32)	0.141*
**Soap near handwashing facility**					
No	254(64.0)	180	74	0.88(0.56-1.39)	0.586
Yes	143(36.0)	105	38	1	1

1 Reference category

* Variables from bivariate analysis of $p<0.25$

Table 4: Factors significantly associated with latrine utilization from multivariable analysis in semi-urban of Alansha area, South Wollo, and January to March, 2019

Variables	Latrine utilization (*N* = 397)		COR (95% CI)	AOR (95% CI)
	Utilized (*n* = 285)	Not utilized (*n* = 112)		
	Frequency	Frequency		
**Household size (persons)**				
1-3	267	98	3.81(1.18-12.30)	3.99 (1.20-13.24)
4-6	13	7	2.60 (0.60-11.31)	3.05 (0.67—13.90)
>6	5	7	1	1
**Presence of primary or secondary school student**				
No	57	42	1	1
Yes	228	70	2.4(1.49-3.88)	2.33 (1.42-3.3.83)
**Years since latrine constructed**				
<2	149	74	1	
≥2	136	38	1.78(1.13-2.80)	1.82(1.12-2.95)
**Cleaning frequency**				
Weekly	64	34	0.83 (0.47-1.48)	1.03 (0.57-1.89)
Daily	78	18	1.93 (1.02-3.67)	2.19 (1.12-4.28)
When dirty	60	23	1.16 (0.62-2.15)	1.33 (0.69-2.54)
Other (rarely)	83	37	1	1

COR, Crude odds ratio; AOR, Adjusted odds ratio; CI, Confidence interval.

**Denotes adjusted odds ratio using 95% confidence interval in multivariable logistic regression analysis.

*Variables included in the multivariable analysis were family size, presence of primary or secondary school student, years since latrine constructed and cleaning frequency of latrine.
