# Supplementary material for: Household latrine utilization and associated factors in semi-urban areas of northeastern Ethiopia
Source: PLoS One. 2020 Nov 12;15(11):e0241270. doi: 10.1371/journal.pone.0241270 (PMC7660512; doi:10.1371/journal.pone.0241270)
Supplement: Attachment — Submitted filename: Latrine review report.docx [file pone.0241270.s006.docx]

Review report (Reviewer # 1 Habtamu Tolera)

The manuscript reports the findings regarding Household Latrine utilization and its association with household family size in semi-urban areas of Alansha, south Wollo, northeastern**,** Ethiopia. This kind of study is much relevant in context of developing countries like Ethiopia. The study has large sample and tried to represent each unit from the study setting Analysis has been made well and the authors have made relevant conclusions/recommendations based on the findings. However, I have some concerns which should be addressed and revised accordingly to get published in PLoS ONE.

**Abstract**

- Page # 2 line 30, under the methods section it was said that “a cross-sectional study was conducted … among **397 households”**. Likewise, on page # 2 line 40 under the same section author (s) also mentioned a total of **401 households** … were assessed ...” Why different figures? This needed to be corrected. On page # 2 line 38 you should delete the unnecessary phrase used in the bracket which is called “**significant at**” because *_Р_*-value is enough to describe the significance of the model high level scholars or readers.

**Background**

- On page # 2 lines 64 and 65 needs you citing source (s). This concern works for all if such cases were there elsewhere across the text.
- On page 4 line 66 and 67 you described that “In 2010, 67 15% of the population still practiced open defecation” Why did not refer recent global report or figure?
- PLoS ONE follows Vancouver style for in text-citation, should be indicated by the reference number in rectangular brackets but authors used APA style across the entire manuscript. Suiting journal’s instructions is vital.
- On page # 4 line 69 you should have to discuss Ethiopian (local) context regarding the prevalence of open defection. Use at least 2016 EDHS report, the proportion of overall population in the country who use open defecation or open field than modern latrine facilities. On page 4 lines 74 and 75 you described that “Lack of latrine utilization has been due mainly to, …” You need to acknowledge or cite original source(s).
- On page # 4 lines 80-82 seems concluding point. Put it under “conclusion section” in the last page of the manuscript.
- Page # 5 Line 95 delete the phrase “recent data from the”.
- On page # 5 Line 100 you need to mention EDHS 2016 as it was the most recent survey than the Mini DHS of 2014. so better to describe like this, “The recent data from the Ethiopia Demographic and Health Survey (EDHS) (2016) indicated that 6% of Ethiopian households…”

**Materials and methods**

- On page # 5 line 116, under a section called study setting “which is located….” is appropriate**.**
- Delete the outcome variable from the sub-section “Study design and outcome variable”. Move your whole discussion about outcome variable and insert before a sub-section “Independent variable”, separately or you can merge both outcome and independent variables into one under a sub-tittle “Variables measured”. Hence, move the whole texts from lines 126 through 134 there.
- Page # 6 lines 129 and 130 you mentioned, “…World Health Organization (WHO) and Joint Monitoring Program (JMP) “signs of use …” . It needs you citing the original source
- Consistency should be kept across the manuscript, e.g. on page 6 line 116 you used *woreda* but on page # 4 line 85 I have seen “In Denbia District” and elsewhere you also used the word “district”, I advise you use either of the two, “*Woreda* “(should be italicized) or “District”
- **Page # 9 line 178, you mentioned that,” …**Environmental Health” use lower case. Page #9, line 180, rewrite the sentence like this “The principal investigator provided a training that took the whole one day…”
- **Page # 9 lines 191 through 193** does not convey a clear message for readers. Please rewrite this sentence
- **On page # 10 under a section Independent variable you missed to list the** Households wealth status distributions across five categories: poorest, second, middle, fourth and highest should be described as an exposure variable. you need to discuss the issue here as well
- On page # 10 line 208, a kind of ambiguity happens there, so delete “other” and use instead “Ash” or use “other” and define what it features in bracket
- Ethical consideration needs citing of ECR’s protocol number of the approval letter.
- Move the sentence on page # 11 from lines 216 through 218 to the last section of this paragraph.

**Results**

- Rewrite line 239 like this**, “**In this study, of the total 401 participants,”, Line 240 like this, “Out of this, the majority (71.8%),...”. Line 241 like this, “Across gender, of the total 397 respondents,”. Line 243 lie this, “who were Muslims were…”, “other religion followers were…”
- Table caption for Table 1 should be placed in the text immediately after you referenced the table in the in-text citation, in the last paragraph of your respective discussion about the table. This comment works for the remaining tables in the entire manuscript or elsewhere in the text.
- On page # 12, rewrite the sentence from lines 247 through 250. Correct line 252 like this, “Of the total respondents,30.2% of households reportedly showed that they had a cleaning frequency (of latrines) rarely while 24.7% reported that they made every day”. Please line 253 is not clear for me rewrite.

**Discussion**

- On page 14 line 282, rewrite like this, “in this study, the rate of latrine facility utilization…”. Line 283 and 284 like this, “elsewhere in other parts of Ethiopia such as...”. Line 285 through 290 is too long sentence. Please keep it succinct and to the point.
- Try to rewrite a sentence from line 292 through 295 like this, After adjusting for all variables, a socio-demographic factors in a form of households with small family size of 1-3 members were significantly associated with latrine facility utilization as opposed to households with larger family members (4 person or more), which is consistent with studies in southeastern zone of Tigray(), and in Hawassa ( )
- **Lines 297 through 299 is n**ot clear, rewrite this paragraph.

**Conclusion**

In conclusion, there is no need to make discussion, just write the major conclusions and what recommendations they could made for policy makers. Your conclusion under abstract section is better stated than the one that was found at the last page of the manuscript. **Move the concluding points under Abstract section to your Major “Conclusion” section in the last page of the manuscript.**

**Acknowledgement**

- In PLoS ONE, you acknowledge funders not under Acknowledgment section but under funding section during online submission.

**Reference list**

- Change reference lists into Vancouver style please. Avoid capitalization problems as well. Do according to the Journal’s guideline

Overall, some editorial works are here in the manuscript. It should be made more concise and clearer in each section. They should check and revised thoroughly.
