# Supplementary material for: Household latrine utilization and associated factors in semi-urban areas of northeastern Ethiopia
Source: PLoS One. 2020 Nov 12;15(11):e0241270. doi: 10.1371/journal.pone.0241270 (PMC7660512; doi:10.1371/journal.pone.0241270)
Supplement: Attachment — Submitted filename: Reviewer comments on PONE-D-20-01859.docx [file pone.0241270.s004.docx]

To be accepted for publication in PLOS ONE, research articles must satisfy the following criteria:

1. The study presents the results of original research.

Yes
2. Results reported have not been published elsewhere.

Yes
3. Experiments, statistics, and other analyses are performed to a high technical standard and are described in sufficient detail.

No
4. Conclusions are presented in an appropriate fashion and are supported by the data.

Yes
5. The article is presented in an intelligible fashion and is written in standard English.

Yes
6. The research meets all applicable standards for the ethics of experimentation and research integrity.

Yes
7. The article adheres to appropriate reporting guidelines and community standards for data availability.

Yes

**Decision of reviewer:**

This manuscript accepted with major comments.

**Comments on Manuscript:**

Overall this is a good study but needs to be reviewed and toned down by the authors for the following checks:

Title:

The authors may revise the language of the title/s to improve readability.

Author may consider writing the title as “Household Latrine Utilization and Its Association with Household Family Size in Semi-Urban Areas in Ethiopia”

Abstract:

1. Author reported a different number of households in method and result of the abstract. In line 30, number of households mentioned 397 but in line 40, it was mentioned 401. Later on, in line 151 final sample sizes mentioned as 401. I would suggest checking the number and making it clear.
2. In line 41, need a space between 285 and (71.8%).
3. In line 42 put a coma (,) instead of full stop (.) after the value 3.99
4. In line 43, need a space between constructed and ≥2 years. Author made the same error in line 240-242 as well as in many lines of the manuscript and in the table of result. I would suggest checking for the same error throughout the manuscript.

Materials and Methods

5. In line 176, author may consider to write “spot check observation” instead of “observation”.
6. In line 207, what do author mean by “kebele”? Make it clear.

Results

I would recommend the author to re-write the result section including the results Tables (Table1 to table 4). Author need to work on the following points:

7. Report the prevalence and keep the number within bracket, eg 71.8% (285). Make the changes throughout the result section. In addition, remove the decimal if the prevalence more than 10%. Author should report the Bivariate result in the result section.
8. In line 247, rewrite the result to improve readability.
9. In line 261, check the value of 95% CI. A proof reading is must for the author, specially in the result section.
10. The title of the tables is too long. I would recommend the author to make it short. Better to remove the location and duration from the title, eg. “Table 1: Socio-demographic characteristics and bivariate analyses with latrine utilization in semi-urban area in Ethiopia”.
11. Author may consider bold the significant results in the tables.
12. In line 423-424 (Table 1), I would recommend the author to report prevalence for the column utilized and not utilized. Same recommendation for the rest of the tables. Without prevalence it’s difficult to check the bivariate results.
13. In line 454-455 (Table 3), author mentioned “n (%)” in 3^rd^ and 4^th^ column but only report the frequency. I would recommend to report both frequency and %

Discussion:

14. In line 278, Author should remove number (prevalence or 95% CI) form the discussion section. It’s not a good practice to report the result in the discussion.
15. Author should work on the discussion depending on the changes of result section.
