# Supplementary material for: Household latrine utilization and associated factors in semi-urban areas of northeastern Ethiopia
Source: PLoS One. 2020 Nov 12;15(11):e0241270. doi: 10.1371/journal.pone.0241270 (PMC7660512; doi:10.1371/journal.pone.0241270)
Supplement: Attachment — Submitted filename: Reviewer comments on PONE-D-20-01859.docx [file pone.0241270.s007.docx]

**Decision of reviewer:**

This manuscript is accepted.

**Comments on Manuscript:**

It is really very nice to see the progress of the manuscript. The author worked very hard to improve the manuscript and addressed all of the comments that I had properly. It is now very well developed.

Title:

The author revised the title and now it looks great.

Abstract:

The author addressed all of the comments that I had in the abstract.

Materials and Methods

My comments in this section addressed properly.

Results

The author made a great progress in the result section. I don’t have any additional comments on it.

Discussion:

This section is very well written. The way to present the internal and external validity looks great.
